# Signaling and other functions of lipids in autophagy: a review

**DOI:** 10.1186/s12944-020-01389-2

**Published:** 2020-09-30

**Authors:** Alejandro Soto-Avellaneda, Brad E. Morrison

**Affiliations:** 1 Biomolecular Sciences Graduate programs, Boise State University, Boise, ID 83725 USA; 2 Department of Biological Sciences, Boise State University, Boise, ID 83725 USA

**Keywords:** Autophagy, Lipids, Mammalian target of rapamycin, Peroxisome proliferator-activated receptor, Fatty acids, Phospholipids, Sphingolipids

## Abstract

The process of autophagy is integral to cellular function. In this process, proteins, organelles, and metabolites are engulfed in a lipid vesicle and trafficked to a lysosome for degradation. Its central role in protein and organelle homeostasis has piqued interest for autophagy dysfunction as a driver of pathology for a number of diseases including cancer, muscular disorders, neurological disorders, and non-alcoholic fatty liver disease. For much of its history, the study of autophagy has centered around proteins, however, due to advances in mass spectrometry and refined methodologies, the role of lipids in this essential cellular process has become more apparent. This review discusses the diverse endogenous lipid compounds shown to mediate autophagy. Downstream lipid signaling pathways are also reviewed in the context of autophagy regulation. Specific focus is placed upon the Mammalian Target of Rapamycin (mTOR) and Peroxisome Proliferator-Activated Receptor (PPAR) signaling pathways as integration hubs for lipid regulation of autophagy.

## Introduction

**Fig. 1:**
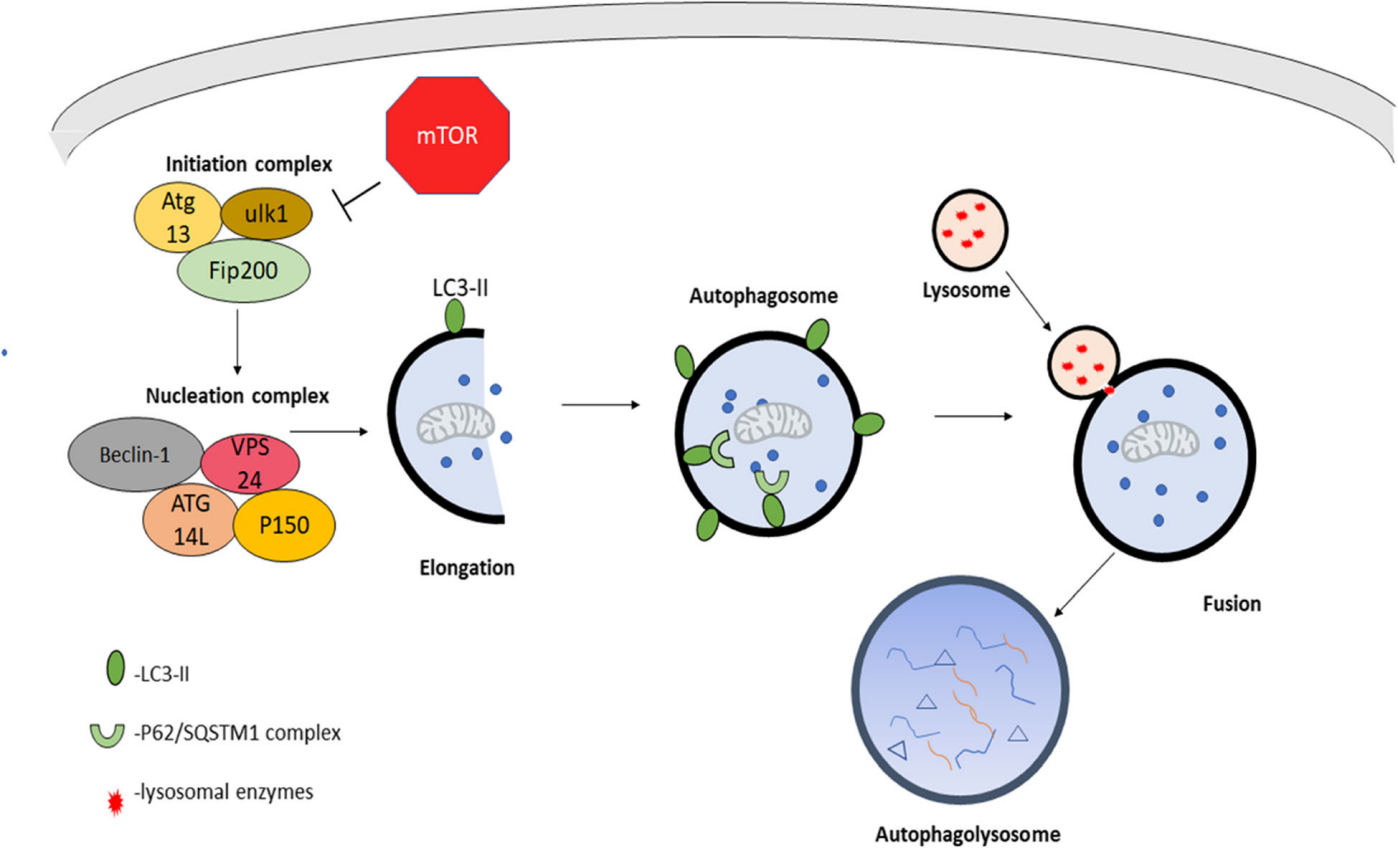
Autophagy mechanism. Autophagy is a cellular mechanism by which metabolites, organelles, proteins, and protein aggregates are enveloped by a vesicular membrane to form an autophagosome. The autophagosome is trafficked to a lysosome where fusion occurs, and lysosomal degradative enzymes break down the cargo

Autophagy is a process by which proteins, organelles, and metabolites are broken down and turned over often as a response to starvation or as a means to protect the cell from damage. Autophagy pathways come in three forms, macroautophagy, microautophagy, and chaperone-mediated autophagy [1]. Of these, macroautophagy is the best characterized and most well understood. Macroautophagy (hereafter referred to as autophagy) was originally studied in yeast and involves the formation of lipid vesicles known as autophagosomes that engulf cargo to be degraded. Once formed, the autophagosome is trafficked to a lysosome and a fusion event occurs resulting in the degradation of the cargo within the autophagosome (Fig. 1) [1].

Autophagy is delineated into key events: initiation, nucleation, elongation and formation of a mature autophagosome, fusion of the autophagosome with a lysosome, and degradation of cargo. The initiation of autophagy is tightly regulated by the mTOR complex 1 [2]. When the cell is in a nutrient-rich state, mTORC1 is active and autophagy is suppressed, however, during nutrient-poor conditions, mTOR is inhibited which allows for the formation of Unc-51 like kinase (ULK) initiation complex composed of ULK kinases, autophagy-related protein 13 (Atg13), Autophagy related protein 101 (Atg101), and RB1-inducible coiled-coil protein 1 (FIP200) [3]. Furthermore, ULK-1 also activates a second complex composed of Beclin1-vacuolar protein sorting protein 34 (VPS34)-autophagy related protein 14 (Atg14L)-P150, which produces phosphatidylinositol-3-phosphate (PI3P) (Fig. 1) [4]. This complex is responsible for autophagic vesicles budding from the endoplasmic reticulum and forming a structure known as an omegasome. In mammals, this is the site responsible for the nucleation of autophagosomes [5]. Next, phosphatidylethanolamine (PE) is conjugated to microtubule-associated light protein light chain 3 (LC3) by autophagy-related protein 7 (Atg7) and autophagy-related protein 3 (Atg3), which are ubiquitin-like conjugating enzymes. Then the conjugated PE-LC3 is inserted into the autophagosome membrane [6]. In addition, autophagy-related protein 12 (Atg12) is conjugated to autophagy-related protein 5 (Atg5) by Atg7 and autophagy-related protein 10 (Atg10) also in a ubiquitin-like manner [7]. Atg12-Atg5 interacts with autophagy-related 16 like protein (Atg16L) and promotes elongation [8]. Meanwhile, cargo is selected by ubiquitination and interaction with cargo receptor proteins p62/ Sequestome 1 (sqstm1). Cargo bound to p62 then binds to the p62 interacting regions of LC3 [9]. After the cargo is selected, the autophagosome matures by disassembling the autophagy-related proteins from the outer layer with the help of myotubularin 3 (MTMR3), a PI3P phosphatase [10]. Once matured, the autophagosome will fuse with early and late endosomes as well as with lysosomes this is mediated by Rubicon, UV resistance-associated gene (UVRAG), Ras-related protein 7 (Rab7), snap receptor proteins (SNAREs), and Lysosome-associated membrane glycoproteins (LAMPs) [11–13]. Once fusion with a lysosome is complete, the cargo is degraded. In mammals, lysosomal hydrolases break down cargo. Beneficial components, such as amino acids are then returned to the cytosol via amino acid efflux proteins such as vacuolar amino acid transporter 3 (Avt3) and vacuolar amino acid transporter 4 (Avt4) [14].

## Lipid signaling directs autophagy

While autophagy has been studied extensively over the years, the role of lipids in this process is underrepresented. Historically, working with lipids has presented a challenge, leading to an emphasis on work that primarily focused upon protein contributions. However, recent advances in both mass spectrometry capabilities and methodologies have spurred considerable progress in the study of lipids. For example, lipophagy, the targeted breakdown of lipid droplets by autophagic pathways, is currently being studied in the context of non-alcoholic fatty liver disease, aging, and cancer. It is becoming more apparent that lipids play a prominent role in autophagy. mTOR, the master regulator of cell growth, metabolism, and autophagy is itself a part of a signaling cascade in which lipid phosphoinositides are involved. In addition, Peroxisome Proliferator-activating factors (PPARs), are nuclear receptors that respond to lipid signals and have been implicated in the control of autophagy and autophagy-related genes. For all of these reasons, this review seeks to provide a comprehensive overview of the growing field of lipid signaling. In the subsequent sections of this article, we discuss the different lipid signaling pathways known to regulate autophagy and their implications in disease states.

## Impaired autophagy in human disease

Autophagy is of considerable interest as a potential target for treatment in many diseases that include cancer, muscular disorders, and neurodegenerative disease. The fundamental role for organelle, particularly mitochondria, and biomolecule turnover by autophagy provides a broad influence of this process in cellular physiology. In addition, autophagy is the only known cellular process for removing protein aggregates making the study of this process of considerable interest in protein aggregation disorders which coincide with numerous neurodegenerative diseases. Therefore, understanding and developing tools to manipulate autophagy could yield widespread therapeutic benefits.

Due to its regulation by mammalian target of rapamycin (mTOR), autophagy is intimately involved in growth, cell death, and cytoprotective processes. As a result, there is great interest in harnessing this process in the context of cancer. In the early stages, suppression of autophagy is believed to facilitate the uncontrolled growth [15]. In later stages, cells may require increased autophagy in low-oxygen and low-nutrient conditions, such as those seen in tumors [16]. Autophagy also can protect tumors from ionizing radiation by helping to remove damaged organelles and proteins [17]. Dysfunction in the phosphatidylinositol-3-kinase (PI3K)-protein kinase B (Akt)-mTOR pathway has been commonly seen to result in altered autophagy. This pathway, when active, suppresses autophagy and uses lipid signaling molecules such as phosphatidylinositol-3,4,5-triphosphate (PIP_3_) as key signal transducers [18]. Mutations in phosphatase and tensin homolog (PTEN), a phosphatase that antagonizes PI3K and causes positive regulation of autophagy, result in aberrant inhibition of autophagy that has been associated with excessive growth and tumor formation [19]. Another common mutation in cancers that leads to autophagy dysfunction is Beclin-1. A high percentage of human breast, ovarian, and prostate cancers have a heterozygous mutation in this gene. Beclin-1 is a part of the initiation complex responsible for activating lipid kinases required for the formation of autophagosomes. In breast carcinoma cell line MCF7, it has been established that Beclin-1 expression is below detectable limits, and transfection of the Beclin-1 gene upregulates autophagy [15]. Studies have also shown that mice with a heterozygous deletion of Beclin-1 are more susceptible to developing tumors [20, 21]. This is further evidence of the role of beclin-1 and autophagy play in cancer.

Autophagy has also been implicated in muscular disorders. It is common for autophagy to play an important role in post-mitotic cells, such as muscle cells and neurons due to the potential for damage from the accumulation of dysfunctional or toxic molecules, protein, or organelles. Vacuolar myopathy is a type of muscular disease in which the structure of lysosomes is abnormal either from a deficiency in lysosomal enzymes or a deficiency in lysosomal membrane proteins [22]. Therefore, it is not surprising that diseases in which lysosomal function is affected also result in altered autophagy. In fact, an accumulation autophagosomes is typically required to diagnose vacuolar myopathies [23]. In addition, an autophagy-related gene has been associated with a vacuolar myopathy known as Danon’s disease. In Danon’s disease, mutations in lysosome-associated membrane protein 2 (LAMP-2) have been identified [23]. LAMP-2 is a lysosomal membrane protein whose function is still not fully understood. However, studies in which the Lamp2 gene is deleted in mice result in a Danon’s disease-like phenotype and accumulation of autophagosomes [23].

Autophagy has long been thought to play an important role in neurodegenerative disorders. A prominent hallmark of these diseases is the accumulation of protein aggregates associated with neuronal loss in the brain. Noted examples include ɑ-Synuclein Lewy bodies in Parkinson’s disease, Tau neurofibrillary tangles in Alzheimer’s disease, Superoxide dismutase 1 (SOD1)-mediated aggregates in Amyotrophic Lateral Sclerosis, and mutant Huntingtin protein aggregates in Huntington’s disease [24–28]. It is speculated that these aggregates may be substrates for autophagy. It is also thought that in these disease states, autophagy is disrupted. Several proteins have been identified and are linked to dysfunction in various steps of autophagy in each of these diseases. For example, in Alzheimer’s disease, autophagy induction is disrupted by reduced expression of Beclin-1 [29]. In Parkinson’s disease, an overexpression of ɑ-Synuclein causes the inhibition of GTPase Rab1. This inhibition is responsible for the mislocalization of autophagy-related protein 9 (Atg9), a protein involved in the formation of autophagosomes [30]. Also, PTEN induced kinase 1(PINK1) and Parkin are proteins involved in the recognition of damaged mitochondria normally targeted for degradation mitophagy. Loss of function mutations in these proteins can prevent the necessary destruction of damaged mitochondria through autophagy resulting in cell death [31, 32]. In addition, the *park9* gene encodes lysosomal type 5 P-type ATPase (ATP13A2). Autosomal recessive mutations in the *park9* gene result in levodopa-responsive early-onset Parkinson’s Disease. This loss of function mutation is responsible for aberrant expression of zinc transporters and impairment in the ability for Zn 2+ to enter lysosomal vesicles resulting in an induction of reactive oxygen species and impairment of mitochondrial function [33]. In Huntington’s disease, mutant huntingtin protein is known to affect several stages in autophagy [32]. Many research groups have reported changes in the expression of mRNA corresponding to genes in the autophagic pathway [34]. In addition, a polymorphism in the ATG7 gene has been linked to early-onset Huntington’s Disease [35].

## Phospholipids, sphingolipids, and mTOR signaling

Phosphoinositides are a class of phospholipids derived from phosphatidylinositol, which is found in the inner layer of the cell membrane and are commonly used by the cell as signaling molecules [36]. They play a major role in the regulation of autophagy through phosphorylation and dephosphorylation at the 3,4 and 5-hydroxyl positions of the inositol ring. They control the pathway that directly activates or deactivates mTOR [37]. mTOR itself is a master regulator of growth, anabolic processes, and autophagy. Generally, mTOR is activated in response to insulin, other nutrients such as amino acids or triglycerides, and growth factors. When active, mTOR promotes growth and suppresses autophagy. In response to starvation, the cell inhibits mTOR, and autophagy is promoted [2].

**Fig. 2:**
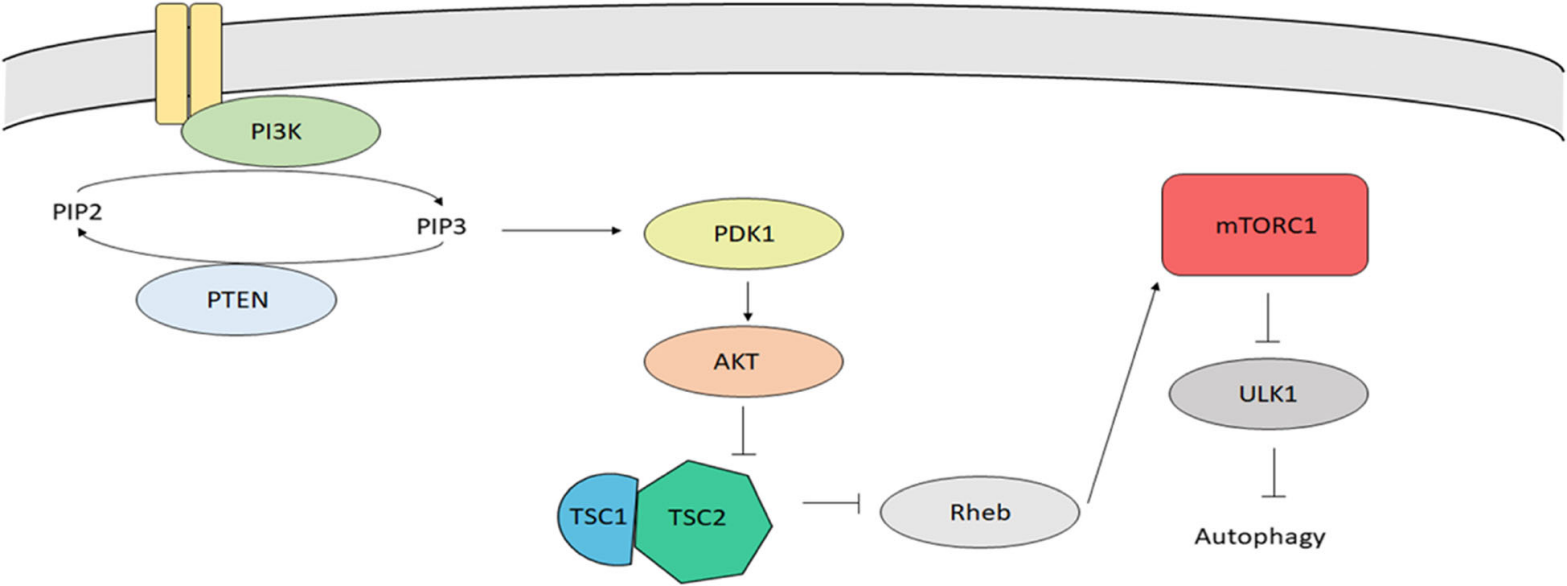
PI3K-mTOR autophagy pathway utilizes PIP3 lipid signaling. PI3K converts the lipid PIP2 to PIP3. PIP3 mediates the phosphorylation of PDK1 causing the activation of AKT. AKT inhibits the activation of the TSC1/2 complex by phosphorylating TSC2. The inhibition of the TSC1/2 complex results in inhibition of Rheb GTPase which in turn activates mTORC1. MTORC1 inhibits the activation of the ULK activation complex leading to an inhibition of autophagy. Inversely, phosphatase activity of PTEN converts PIP3 to PIP2 which suppresses the activation of PDK1 and downstream AKT. Inhibited AKT cannot suppress the TSC1/2 complex allowing Rheb GTPase to remain active. An active Rheb results in inhibition of mTORC1 and an activation of the ULK1 complex

The canonical signaling pathway that controls autophagy through PIP_3_. The pathway begins as a response to insulin, other nutrients, or growth factors [38]. Phosphoinositide 3-kinases convert phosphatidylinositol 4,5- bisphosphate (PIP_2_) to PIP_3_. PIP_3_ activates phosphoinositide-dependent kinase-1 (PDK1) which in turn phosphorylates Akt [39, 40]. Akt then phosphorylates tuberous sclerosis 2 (TSC2) which results in the inhibition of tuberous sclerosis 1/2 (TSC1/2) complex [41]. When inhibited, TSC1/2 cannot activate Rheb GTPase activity permitting activation of mTOR. When bound to GTP, Rheb mediates the activation of mTOR complex 1 (mTORC1) which, in turn, inhibits autophagy [42]. Activated mTORC1 inhibits autophagy by inhibiting the ULK1 initiation complex. Pro-autophagy signals result in ULK1 dissociation from mTOR and autophagy initiation is facilitated (Fig. 2) [43].

PI3P also plays an integral role in the process of autophagy by interacting with VPS34 [44]. Originally identified and studied in yeast, VPS34 is a class III phosphatidylinositol 3 kinase. In yeast, VPS34 forms one of two complexes. VPS34 complex-I is composed of VPS34, VPS30/ATG6, VPS15, and ATG14 and is implicated in the initiation of the pre-autophagosomal structure (PAS) [45, 46]. In Mammals, VPS34 is thought to play a similar role in autophagy initiation. However, it has been difficult to study in mouse models since pan knockouts of VPS34 are embryonically lethal, and there are no inhibitors specific to VPS34 necessitating the use of low specificity inhibitors, such as wortmannin or 3-MA [47]. Conditional knockout studies using cultured mice embryonic fibroblasts have shown that VPS34 is required for the formation of autophagosomes [48]. In addition, VPS34 is involved with mTOR regulation of autophagy. Studies using mice embryonic fibroblasts have shown that mTORC1 must be inactivated for the VPS34 initiation complex to be active and that mTORC1 can inhibit the phosphatidylinositol 3-kinase activity of this complex by phosphorylating ATG14 [48]. PI3P is also a component of the autophagosome. It has been observed to be enriched in the concave surface of early phagophores [49]. Because of this, PI3P is thought to facilitate the expansion and sealing of autophagosomes.

In addition to its function in the autophagosomal membrane, PI3P is thought to mediate selected cargo capture via its interaction with autophagy linked FYVE protein (Alfy), a nuclear scaffold protein with a FYVE domain that binds PI3P [50]. In the autophagic process, Alfy interacts with Ath5 and P62 through its WD40 domain and with PI3P through its FYVE domain. The high amount of PI3P in the inner membrane of autophagosomes is believed to act as a dock for this Alfy/Atg5/P62 complex in the selective engulfment of protein aggregates that results in aggregate clearance known as aggrephagy [51].

Metabolites formed from the breakdown of phospholipids are also involved in autophagy. Phosphatidic acid is formed by the breakdown of phosphatidylcholine into choline and phosphatidic acid by Phospholipase D [52]. Phosphatidic acid plays a role in autophagy by inducing membrane curvature due to its cone shape. In addition, Phosphatidic acid is formed as a result of an absence of nutrients and serves as an inhibitor of mTORC1 thus acting as a positive regulator of autophagy [53]. Phosphatidic acid can also be converted into diacylglycerol by the Phosphatidic Acid Phosphatases which has other autophagy regulating properties [54]. Diacylglycerol modulates autophagy by activating Protein Kinase C which induces autophagy by disrupting the B-cell lymphoma protein 2 (Bcl-2)-Beclin-1 complex via c-Jun N terminal kinase (JNK) and Nicotinamide adenine dinucleotide phosphate (NADPH) oxidase [54].

Sphingolipids are a class of lipids involved in several processes ranging from apoptosis to cell proliferation to differentiation, inflammation, and autophagy [55]. In autophagy, two sphingolipids have been found to play sizeable roles, ceramide and sphingosine − 1-phosphate. Ceramide activates autophagy by inhibiting Akt and resulting in an inactivation of mTOR and an upregulation of Beclin-1 function [56]. Sphingosine-1-phosphate is formed by the hydrolysis of ceramide into sphingosine followed by its phosphorylation by Sphingosine Kinases 1 and 2 [57]. Sphingosine Kinase 1 is activated by starvation signals and drives the formation of sphingosine-1-phosphate [57]. In addition, sphingosine kinase 1 inhibits mTORC1 independently of Akt while upregulating beclin-1 expression, ultimately promoting cell survival [57].

## Signaling through the PPAR family

**Fig. 3:**
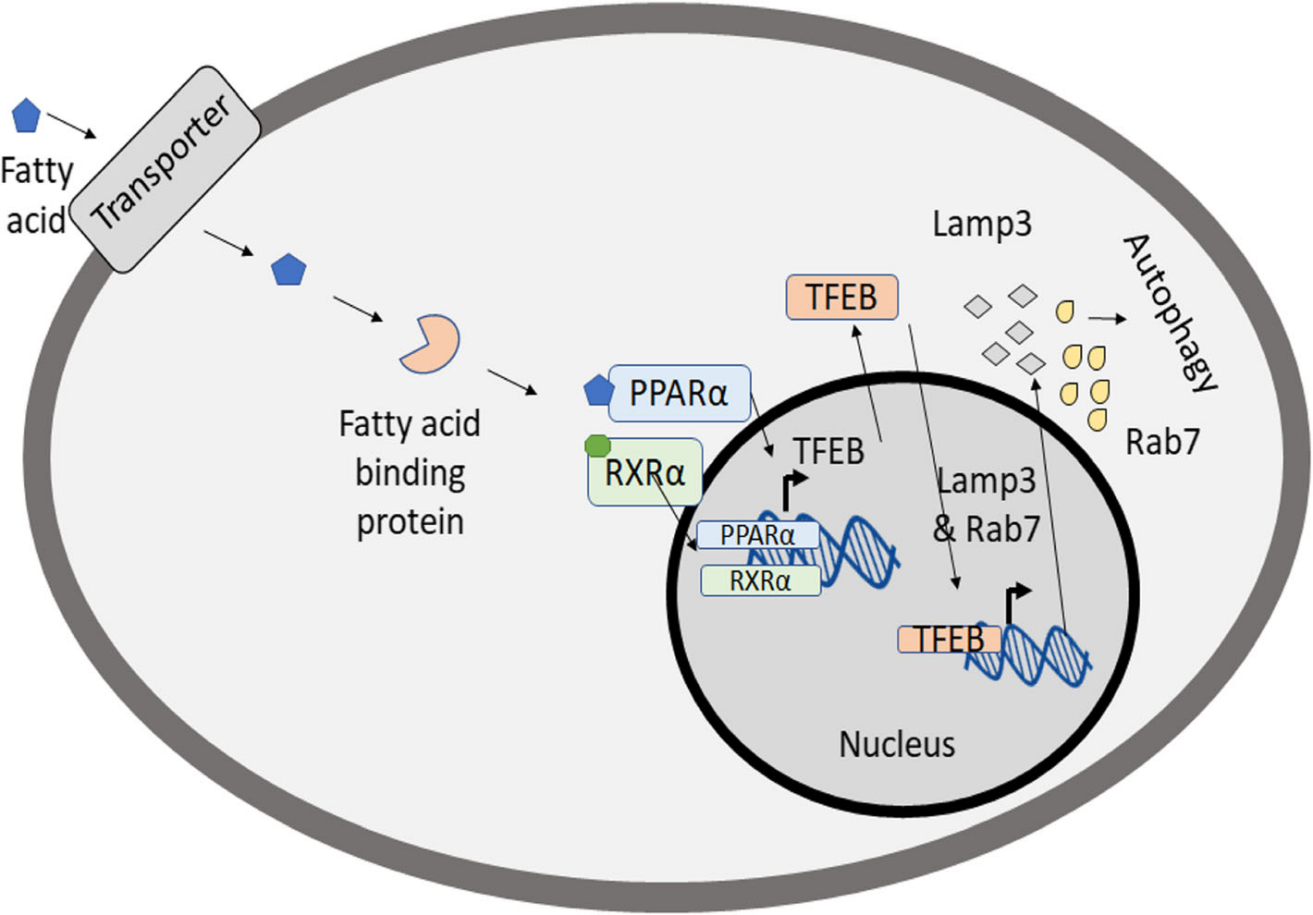
Overview of PPAR signaling and mechanism for PPARα-mediated autophagy activation in innate immune system**.** Initially, lipid molecule enters the cell and is quickly bound by a fatty acid binding protein. The fatty acid binding protein transports the lipid to a PPAR which in turn activates a corresponding RXR. The PPAR-RXR complex crosses into the nucleus and facilitate expression of required genes. In the case of tuberculosis infection, PPARα upregulates the expression of TFEB which, in turn, drives the expression of autophagy related genes, LAMP3 and RAB7 thus stimulating autophagy

PPARs are a family of nuclear receptor proteins that act as transcription factors. There are 3 isoforms of PPARs in mammals, PPARα, PPARδ, and PPARγ [58]. All 3 isoforms of PPARs must bind with a Retinoid Receptor X (RXR) and a lipid ligand in order to act as transcription factors (Fig. 3) [59]. Generally, they have been reported to bind to oleic acid, linoleic acids, linolenic acids, prostaglandins, eicosanoids, and oxidized lipids with the help of fatty acid-binding proteins which bind lipophilic ligands in the cytoplasm and shuttle them to their target PPAR [60, 61]. PPARs bind to their ligands through the ligand-binding domain (LBD). These domains consist of 12 α-helices arranged into an ‘antiparallel helix sandwich’ and a three-stranded antiparallel β-sheet. The ligand-binding site is located in the core of the ligand-binding domain that is formed by helices 3,5,7,11, and 12. The cavity formed by these helices is T-shaped [62]. In order for ligands to bind to PPAR-α or PPAR-γ, they must be able to form a U-shaped conformation, and to bind to PPAR-delta ligands must form an L-shaped conformation [63]. All PPARs isoforms have been shown to modulate autophagy in the context of different diseases and cellular responses.

PPARα is primarily expressed in the liver, brown adipose tissue, heart, and kidney. It promotes uptake and catabolism of fatty acids by helping to express fatty acid transport and binding genes [58, 64]. It has been thought to be involved in the innate immune response during mycobacterium infection [65]. In studies with tuberculosis infected bone-derived macrophages, PPARα was shown to stimulate autophagy and autophagosomal maturation, while suppressing inflammatory responses. It was determined that following PPARα activation, Transcription Factor EB (TFEB) was activated and a series of autophagy and lysosomal genes were expressed such as LAMP3 and Rab7 [65]. Based on this work, it is thought that, in mycobacterial infections, such as tuberculosis, PPARα is activated and in turn activates TFEB. Together they promote the expression of autophagy-related genes that stimulate autophagy (Fig. 3).

PPAR δ has high expression levels in the colon, small intestine, liver, heart, lung, and brain. It plays an important role in diseases such as diabetes, obesity, atherosclerosis, and cancer [58, 64]. This is especially poignant because there are great efforts in exploiting autophagy as possible treatments for cancer and diabetes. Studies in mice cells have shown a marked decrease in autophagic markers associated with the knockout of PPAR δ suggesting its involvement in autophagy [66].

**Fig. 4:**
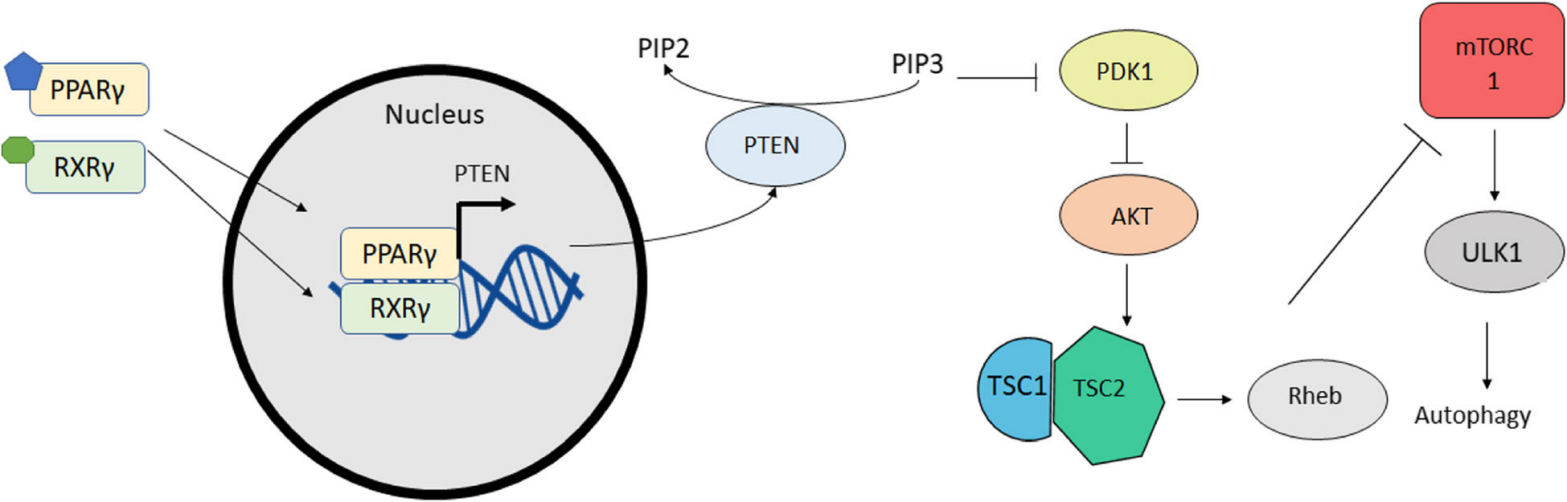
PPARγ mediated activation of autophagy. PPARγ promotes the expression of PTEN. High amounts of PTEN lead to lower concentrations of PI(3,4,5)P3. Less PI(3,4,5)P3 inhibits the activation of PDK1 and ultimately results in inhibition of mTORC1 which causes an activation of autophagy

Finally, PPARγ is expressed in adipose tissue, the intestines, and macrophages. It is usually involved in fatty acid storage, glucose uptake, and adipogenesis [58, 64]. Because of its role in controlling the availability of nutrients, there has been an interest in targeting it as a treatment for cancer. In Colorectal cancer, studies with Caco-2, a common colorectal cancer cell line, have shown that activation of autophagy occurs following treatment with PPARγ agonist rosiglitazone [67]. In addition, inhibition of autophagy with 3-MA was observed to induce the expression of PPARγ. PPARγ was determined to cause the induction of PTEN, an antagonist to PI3K which dephosphorylates and reduces the concentration of PIP_3_ [67]. This results in the overall inhibition of the mTOR pathway and induces autophagy. In the context of breast cancer, PPARγ has also been implicated to modulate autophagy. Activation of PPARγ by agonist troglitazone was shown to induce autophagy in MDA-MB231 cells as determined by the measurement of acidic vesicular organelles by staining with Acridine orange [68]. In addition, studies of constitutively active PPARγ suggest that it is sufficient for the activation of autophagy leading to the belief that autophagy acts to protect cancer cells (Fig. 4) [68].

## Lipid metabolism and autophagy

Autophagy is intricately related to the metabolism of lipids, namely triglycerides because it responds to the presence or absence of nutrients in the cell. Furthermore, it is involved with the breakdown of stored lipids in the cell. Triglycerides are stored in organelles known as lipid droplets. They are used to generate energy, building blocks for membranes, and for lipid signaling [69]. Lipid droplets are broken down for use by the cell via lipophagy. This process is mediated by the GTPase Rab7 in hepatocytes and results in the release of free fatty acids under starvation conditions to be used as fuel in the mitochondria and undergo β-oxidation. Rab7 was shown to mediate the docking of autophagosome to lipid droplets facilitating their catabolism [70]. In addition, Adipose triglyceride lipase (ATGL) is a regulator of lipophagy. When knocked down in hepatocytes, ATGL causes decreased lipophagy. This ATGL signaling has been observed to occur through sirtuin 1. Together, these two proteins drive lipophagy and fatty acid oxidation [71].

Additionally, the breakdown of lipid droplets by lipophagy can be regulated by transcription factors. TFEB mediates the activation of PPAR alpha as a response to nutrient deprivation in order to activate lipophagy [72] Additionally, forkhead homeobox protein O1 (FOXO1) becomes upregulated in nutrient restricted conditions and increases lipophagy of lipid droplets. The FOXO1 mediated lipophagy activation is facilitated by an increased expression of lysosomal acid lipase (LIPA) resulting in a release of free fatty acids through adenosine monophosphate kinase (AMPK)-dependent β-oxidation in adipocytes in nutrient restricted conditions [73].

Conversely, autophagy is linked to the biosynthesis of new triglycerides as well. Not only does autophagy drive the breakdown of lipid droplets, but it is also tied to the metabolic balance of liver triglycerides. Diets low in protein result in reduced expression of autophagy receptor SQstm1 and increases the expression of LC3-II. This correlates to the induction of autophagy. It is speculated that, in the case of low protein availability, autophagy does not catabolize lipids and instead may help triglycerides to accumulate in the liver [74]. Additionally, Perilipin-2, a protein that associates with lipid droplets, has been observed to protect lipid droplets from autophagy. Perilipin-2 has been observed to inhibit lipogenesis and triglyceride production as well as upregulating autophagy when it is depleted in the cell [75].

## Free fatty acids and cholesterol

Free fatty acids have also been implicated in the autophagic pathway. Although they usually act as nutrients, fatty acids can induce cell death when they accumulate in excessive levels in non-adipose cells and tissues. This is known as lipotoxicity and has been observed in diseases such as obesity, diabetes, and non-alcoholic fatty liver disease [76]. As a result, levels of free fatty acids are thought to be regulated inside the cell through lipophagy [69]. Palmitic acid (PA) and its effects on diabetes has been studied in rat pancreatic beta-cell line INS-1 [77]. It was determined to trigger autophagy independently of the mTOR pathway. For instance, autophagy was shown to be promoted by stimulating JNK which leads to phosphorylation of Bcl-2 a resulting in its dissociation from Beclin-1 which in turn allowed for the initiation of autophagy and autophagosome formation [77]. In addition, protein kinase C (PKC) isoforms δ, ɑ, and Θ have also been implicated in PA-mediated autophagy regulation [78].

Studies suggest that in mice embryonic fibroblasts, PA, a saturated fatty acid can induce autophagy [79]. It was reported that palmitic acid was able to increase the amount of LC3, suggesting the induction of autophagy. However, there was no increase in phosphorylation of P70S6K or S6, two downstream proteins in the mTOR signaling pathway [79]. This suggests that PA induces autophagy independent of mTOR. PKCɑ was identified and shown to be involved in the autophagy inducing process. When it was knocked down with siRNA, LC3 detection fell [79]. Furthermore, studies show that while prolonged exposure to PA causes cell death, short term exposure induces autophagy, this suggests that autophagy is an important protective measure against lipotoxicity caused by PA [79].

**Fig. 5:**
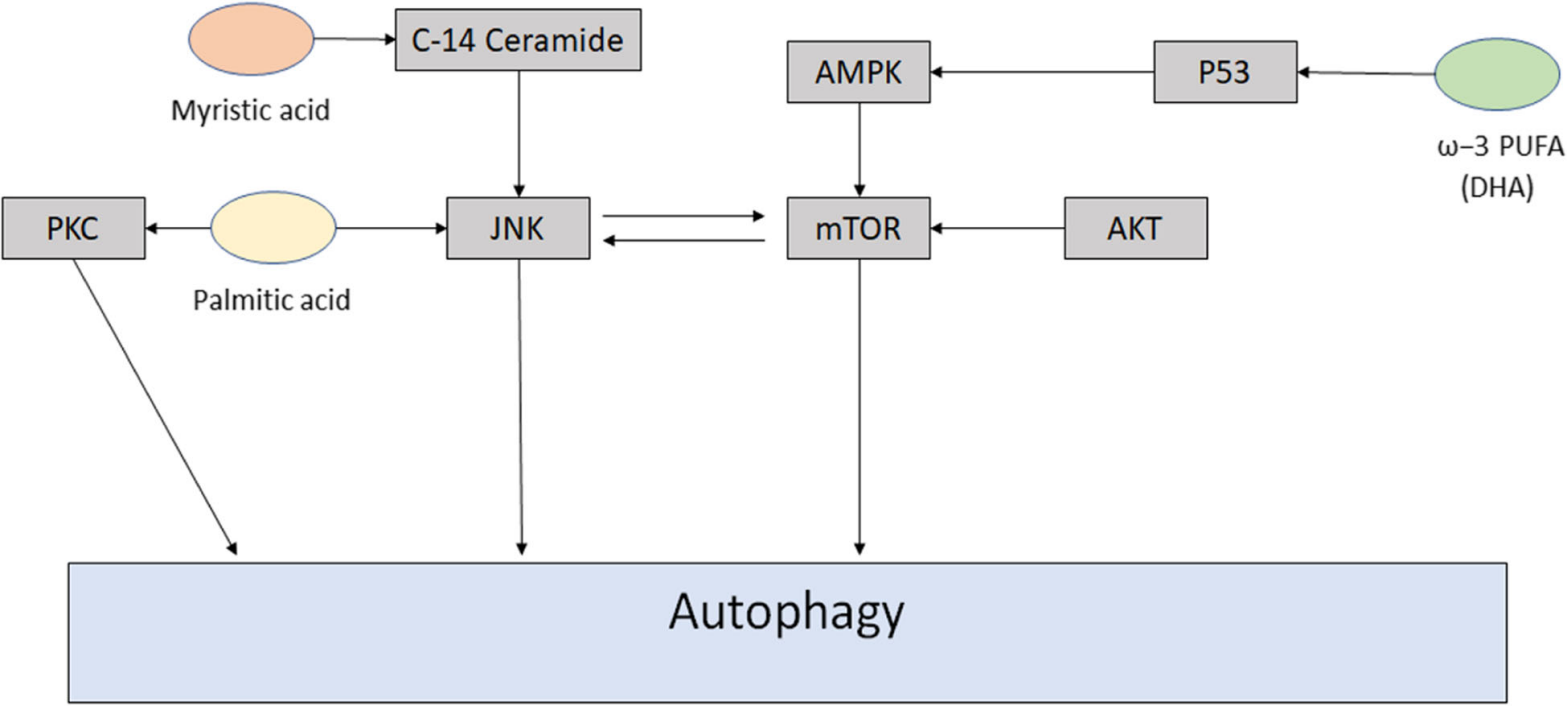
Overview of regulation of autophagy by free fatty acids. Palmitic acid stimulates autophagy by activating JNK and has been shown to interact with PKC isoforms which have been implicated in autophagy regulation. Myristic acid has been shown to upregulate autophagy by producing C-14 ceramide. DHA has been implicated in the P53-AMPK-mTOR pathway resulting in activation of autophagy

PA has been shown to modulate autophagy via a secondary signaling pathway. Its effects have been studied in the context of hepatic steatosis; a condition caused by high amounts of fat in the liver [80]. In hepatic steatosis, high lipid levels cause lipotoxicity. Non-alcoholic steatohepatitis mice were fed a high-fat diet. These mice were shown to exhibit high autophagy mediated by PA [80]. In these studies, autophagy was determined to be regulated by the activation of mitogen-activated protein kinase (MAPK), extracellular signal-regulated kinase (ERK), P38, JNK. Based on these studies, researchers concluded that JNK-1 has a lipo-apoptotic effect while JNK-2 promotes autophagy and has a cytoprotective effect (Fig. 5) [80].

In addition to PA, myristic acid (MA) has been studied for its autophagy regulating effects. Like palmitic acid, MA is a saturated fatty acid commonly used by the body as a nutrient. In the context of autophagy, MA has been found to promote overexpression of Beclin-1 gene BCN1 and increased conversion of LC3-I to LC3-II in mouse cardiomyocytes [81]. MA is thought to work to upregulate autophagy by producing C14-ceramide and upregulating ceramide synthase 5 (Fig. 5) [81].

Omega-3 polyunsaturated acids have been known to play a role in regulating autophagy. The most studied of these is docosahexaenoic acid (DHA). DHA is a component in many of the phospholipids that make up the cell membrane in the brain, skin, and retinal tissue [82]. It has been studied in myocardioblasts and various human cancer cell lines [83–85]. DHA has been implicated in the p53-AMPK-mTOR signaling pathway. It has been shown to work through AMPK to inhibit mTOR and induce autophagy in human cancer cells with wild type p53 [86]..However, In prostate cancer cells with mutant p53, DHA was observed to induce autophagy through the creation of mitochondrial reactive oxygen species that results in the inactivation of AKT and mTOR also resulting in the activation of autophagy (Fig. 5) [87].

Finally, autophagy is related to cholesterol biosynthesis inversely through the mTOR signaling pathway. When cholesterol biosynthesis is inhibited, there is an induction of autophagy. Inhibition of cholesterol biosynthesis by statin drugs such as simvastatin has been linked to autophagy activation via the inhibition of the mTOR signaling pathway in human blood cancer cells. It was determined that the statin drug caused this activation only through cholesterol depletion when the cholesterol depleting agent, methyl-β-cyclodextrin also produced activation of autophagy [88]. Likewise, a deficiency of transmembrane 7 superfamily member 2 (TM7SF2), a positive regulator of cholesterol biosynthesis, results in repression of autophagy. Removal of TMS7F2 causes increases in the expression of fatty acid degradative enzymes, decreased lipid accumulation, and in turn, decreased autophagy in mice exposed to Lipopolysaccharide [89].

## Conclusion and future perspectives

This review aims to outline the current state in the field of lipid regulation of autophagy. To that end, this review has discussed a myriad of ways in which many lipids are involved in the process of autophagy and its regulation. All the lipids discussed along with their functions and effects on autophagy have been summarized in Table 1. Autophagy represents a cellular process that has implications for several important areas of study. Phospholipids and their derivatives have been shown to not only be an important part of the regulation of autophagy through the crucial mTORC1 signaling pathway but also an integral part of the autophagy machinery. These lipids have also been shown to affect a variety of processes by acting through PPARs with the possible implication in human disease. Finally, dietary lipids and cholesterol have been implicated in the regulation of autophagy both through the canonical mTOR pathway and alternative means.

**Table 1:**
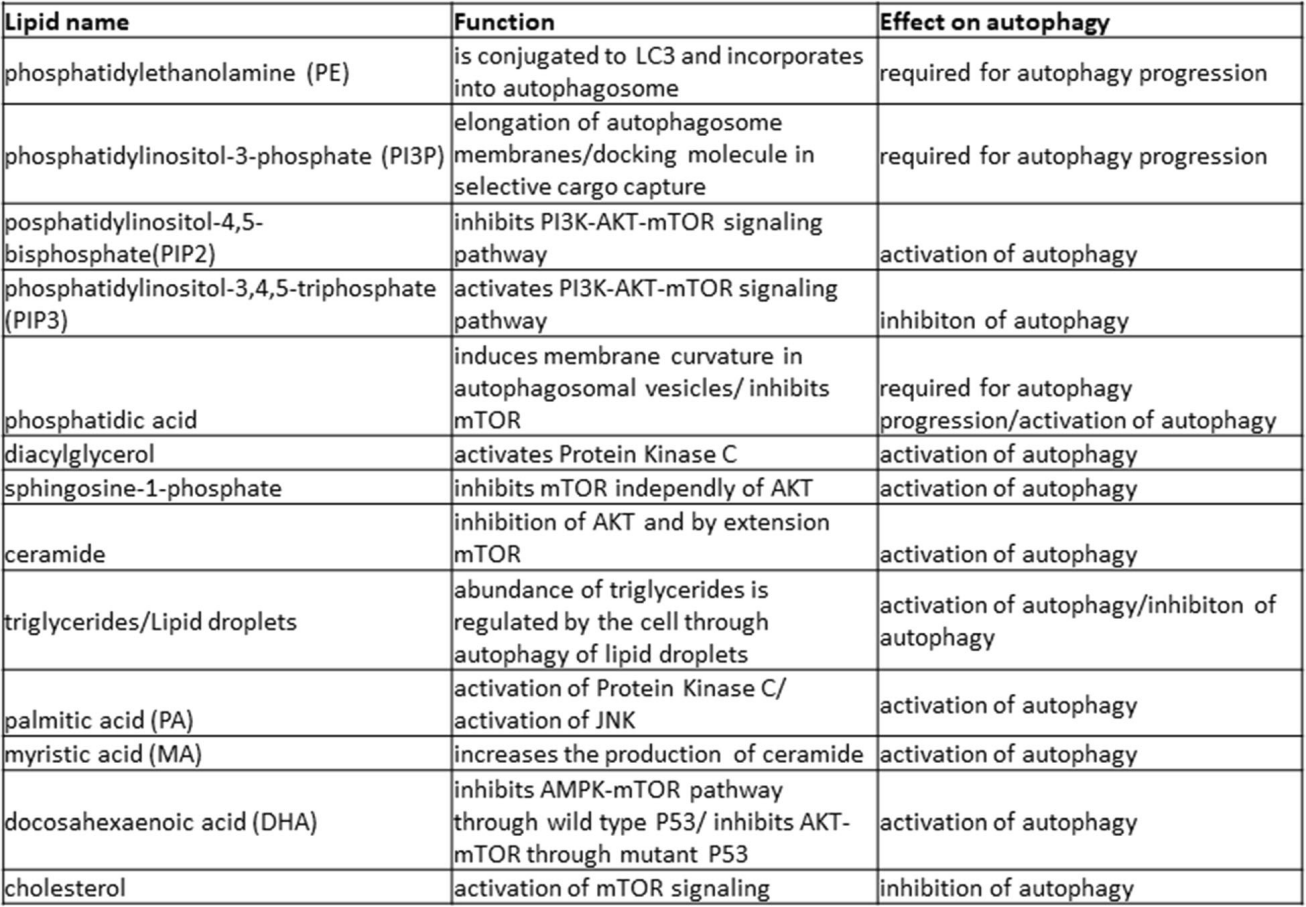
Summary of discussed lipids involved in autophagy

The role lipid molecules play in autophagy represents the potential for many new avenues of research. Understanding it can give us a better, more holistic idea of this process which is central to many cellular functions and disease states. As our understanding of autophagy has grown, its activators and inhibitors have begun to appear as a novel area of drug development. As a result, it is necessary to fill in the gaps in our knowledge concerning lipid signaling. In the specific case of disease, it may be necessary to identify novel lipid molecules involved in autophagy. It may also be helpful to determine if there is a link between the signaling pathways described or if they all act independently. Finally, if autophagy is to be exploited as a potential treatment for disease, it would be necessary to evaluate the effects these lipid molecules and signaling pathways have on cellular functions related to autophagy.

The technology we use to understand autophagy has increased greatly over the past years, and it has allowed our understanding to grow. However, there is still progress to be made especially with respect to the role lipids play in its regulation. For example, mass spectroscopy techniques have significantly improved in recent years allowing for the high throughput analysis and identification of lipids present in a system. Other advances that would facilitate new research could include an improved way of visualizing and even purifying autophagosomes and lysosomes. Current methods utilize immunoprecipitation or density gradient fractionation, both of which are known to result in significant levels of impurities, to isolate these subcellular structures. In conclusion, lipids play an important and diverse signaling role in autophagy regulation, and it is necessary to fully characterize lipid signal transduction pathways to better inform autophagy-based therapies.

## Data Availability

Not applicable.

## Abbreviations

mTOR: Mammalian target of rapamycin
PPAR: Peroxisome proliferator-activated receptor
ULK: Unc-51 like kinase
Atg13: Autophagy-related protein 13
Atg101: Autophagy-related protein 101
FIP200: RB1-inducible coiled-coil protein 1
VPS34: Vacuolar protein sorting protein 34
PE: Phosphatidylethanolamine
LC3: Microtubule-associated light protein light chain 3
Atg7: Autophagy-related protein 7
Atg3: Autophagy-related protein 3
Atg12: Autophagy-related protein 12
Atg10: Autophagy-related protein 10
Atg16L: Autophagy-related 16 like protein 1
Sqstm1: Sequestome 1
MTMR3: Myotubularin 3
UVRAG: UV resistance-associated gene
Rab7: Ras-related protein 7
SNAREs: Snap receptor proteins
LAMPs: Lysosome-associated membrane glycoproteins
Avt3: Vacuolar amino acid transporter 3
Avt4: Vacuolar amino acid transporter 4
PI3K: Phosphatidylinositol 3 kinase
PIP_3_: Phosphatidylinositol-3,4,5-triphosphate
AKT: Protein kinase B
PTEN: Phosphatase and tensin homolog
SOD1: Superoxide dismutase
Atg9: Autophagy-related protein 9
Pink1: PTEN induced kinase 1
ATP13A2: Lysosomal type 5 P-type ATPase
PIP_2_: Phosphatidylinositol-4,5-bisphosphate
PDK1: Phosphoinositide-dependent kinase 1
TSC: Tuberous sclerosis
mTORC1: mTOR complex 1
PAS: Pre-autophagosomal structure
Alfy: Autophagy linked FYVE protein
BCL-2: B-cell lymphoma protein 2
JNK: c-jun terminal kinase
NADPH: Nicotinamide adenine dinucleotide phosphate
RXR: Retinoid receptor X
LBD: Ligand-binding domain
TFEB: Transcription factor EB
ATGL: Adipose triglyceride lipase
FOXO1: Forkhead homeobox protein O1
LIPA: Lysosomal acid lipase
AMPK: Adenosine monophosphate kinase
PA: Palmitic acid
PKC: Protein kinase C
MAPK: Mitogen-activated protein kinase
ERK: Extracellular signal-regulated kinase
MA: Myristic acid
DHA: Docosahexaenoic acid
TM7SF2: Transmembrane 7 superfamily member 2
